# Vitamin D Status among Thai School Children and the Association with 1,25-Dihydroxyvitamin D and Parathyroid Hormone Levels

**DOI:** 10.1371/journal.pone.0104825

**Published:** 2014-08-11

**Authors:** Lisa A. Houghton, Andrew R. Gray, Michelle J. Harper, Pattanee Winichagoon, Tippawan Pongcharoen, Sueppong Gowachirapant, Rosalind S. Gibson

**Affiliations:** 1 Department of Human Nutrition, University of Otago, Dunedin, New Zealand; 2 Department of Preventive and Social Medicine, University of Otago, Dunedin, New Zealand; 3 Institute of Nutrition, Mahidol University, Salaya, Thailand; Faculté de médecine de Nantes, France

## Abstract

In several low latitude countries, vitamin D deficiency is emerging as a public health issue. Adequate vitamin D is essential for bone health in rapidly growing children. In the Thai population, little is known about serum 25-hydroxyvitamin D [25(OH)D] status of infants and children. Moreover, the association between 25(OH)D and the biological active form of 1,25-dihydroxyvitamin D [1,25(OH)]_2_D is not clear. The specific aims of this study were to characterize circulating serum 25(OH)D, 1,25(OH)_2_D and their determinants including parathyroid hormone (PTH), age, sex, height and body mass index (BMI) in 529 school-aged Thai children aged 6–14 y. Adjusted linear regression analysis was performed to examine the impact of age and BMI, and its interaction with sex, on serum 25(OH)D concentrations and 1,25(OH)_2_D concentrations. Serum 25(OH)D, 1,25(OH)_2_D and PTH concentrations (geometric mean ± geometric SD) were 72.7±1.2 nmol/L, 199.1±1.3 pmol/L and 35.0±1.5 ng/L, respectively. Only 4% (21 of 529) participants had a serum 25(OH)D level below 50 nmol/L. There was statistically significant evidence for an interaction between sex and age with regard to 25(OH)D concentrations. Specifically, 25(OH)D concentrations were 19% higher in males. Moreover, females experienced a statistically significant 4% decline in serum 25(OH)D levels for each increasing year of age (*P* = 0.001); no decline was seen in male participants with increasing age (*P* = 0.93). When BMI, age, sex, height and serum 25(OH)D were individually regressed on 1,25(OH)_2_D, height and sex were associated with 1,25(OH)_2_D with females exhibiting statistically significantly higher serum 1,25(OH)_2_D levels compared with males (*P*<0.001). Serum 1,25(OH)_2_D among our sample of children exhibiting fairly sufficient vitamin D status were higher than previous reports suggesting an adaptive mechanism to maximize calcium absorption.

## Introduction

Vitamin D status influences the absorption of calcium and phosphate from the gut and is known mainly for its role in bone health across the lifecycle. During periods of rapid growth in childhood, health consequences of vitamin D deficiency include growth retardation, skeletal deformities and an increased fracture risk later in life [1]. Vitamin D is also associated with non-skeletal health outcomes including modulation of the immune response, reduction of inflammation, insulin production, neuromuscular function, and regulation of cell growth, differentiation and apoptosis [2], [3].

The main source of vitamin D is endogenous production following exposure of 7-dehydrocholesterol in the skin to sunlight (ultraviolet-B irradiation) to produce 25-hydroxyvitamin D [25(OH)D], best known as a marker of vitamin D status. Circulating 25(OH)D is then converted to its biologically active form, 1,25-dihydroxyvitamin D [1,25(OH)_2_D]. Natural food sources rich in vitamin D are scarce. Studies on serum 25(OH)D concentrations have shown a high prevalence (from 15% to 80%) of vitamin D deficiency and insufficiency in several pediatric and adolescents populations [4]–[13]. These findings have been demonstrated not only in higher latitudes, but also in lower latitude countries with ample sunshine. This is possibly due to avoidance of sunlight, indoor lifestyles, clothing coverage and sunscreen use, adiposity, and low dietary intake of vitamin D-rich foods or supplements. Another dietary factor, which may contribute to the development of vitamin D deficiency, is a low calcium diet which, by inducing secondary hyperparathyroidism and elevating 1,25(OH)_2_D, results in increased catabolism of 25(OH)D and a depletion of vitamin D stores [14]–[18].

Thailand, located in the southeastern part of Asia at latitudes between 5°30′ N and 20°30′ N, is amenable to year round vitamin D synthesis. Dietary intake of vitamin D among Thais are low because foods are not fortified with vitamin D. Dietary calcium intake has also been reported to be low in children in Thailand [19], [20]. The Thai diet is predominantly based on rice, vegetables and some meats, and is often low in milk and milk products. The suboptimal calcium content of the diet may be further compromised by a high level of phytate [21]. A recent study of vitamin D status in Thai adults revealed relatively high mean serum 25(OH)D levels with the prevalence of insufficiency [serum 25(OH)D <50 nmol/L] ranging from 3% in the Northeast to 14% in the Bangkok region [22]. Data are currently lacking on the vitamin D status of Thai children. Given the emerging body of evidence on the role of vitamin D in health throughout life, we investigated the vitamin D status of a large sample of school-aged children from northeast Thailand. The specific aims of this study were to characterize circulating levels of serum 25(OH)D, 1,25(OH)_2_D and their determinants including PTH, age, sex, height and body mass index (BMI). Moreover, we examined the impact of age and BMI, and its interaction with sex, on serum 25(OH)D concentrations in these prepubertal children ranging in age from six to 14 years.

## Materials and Methods

### Participants

The present study used endline data from a 31-wk randomized controlled trial (RCT; ClinicalTrials.gov ACTRN12605000341628) assessing the efficacy of a seasoning powder fortified with iron, zinc, iodine and vitamin A for reducing anemia and improving micronutrient status of northeast Thai schoolchildren [23] [24]. The original study was conducted during the school year between June 2002 and March 2003. The participants (261 males, 268 females) were aged 6.0–14.0 y and were randomly selected to participate in the trial from primary schools situated in ten poorest rural subdistricts of the Trakarn Phutphon district, Ubon Rachathani province (latitude 15°N). All of the subdistricts were of low socioeconomic status. For the RCT, children were stratified in each school into four strata: females grades 1–3; males grades 1–3; females grades 4–6; males grades 4–6. Fifteen children were selected randomly from each stratum where possible and in strata with less than fifteen children, all children were selected. To minimize clustering, only one child per household was selected. Children were eligible for the study if they were apparently healthy, and had a hemoglobin concentration greater than 80 g/L. Of the 569 children enrolled in the original study, 25(OH)D, 1,25(OH)_2_D and PTH data were available from 529, 523 and 518 children, respectively, at study endline. Endline samples were collected during the summer season between the months of February and March 2003 (i.e., dry season). The Human Ethics Committee from the University of Otago (New Zealand) and Mahidol University (Thailand) approved the study protocol, and written informed consent was obtained from the children’s parents or guardians.

### Sociodemographic and anthropometric variables

A pretested sociodemographic and health status questionnaire was administered to an adult member of each participating household. Measurements of weight and standing height were taken using standardized techniques by a trained anthropometrist. Z-scores for height, weight and body mass index (BMI) were calculated using the age- and sex-specific WHO Child Growth Standards [25]. Stunted children were defined as those with height-for-age *z*-score less than –2, and underweight and thinness were defined as weight-for-age *z*-scores less than −2 and BMI-for-age *z*-score less than −2, respectively.

### Blood sample collection and biochemical assessment

Morning, nonfasting peripheral venipuncture blood samples were drawn by trained nurses into trace element-free evacuated tubes (Becton Dickinson, Franklin Lakes, NJ), centrifuged (2500 X g, 10 min, room temperature) and serum was stored at −70°C.

Serum 25(OH)D_2_ and 25(OH)D_3_ were determined in-house using isotope-dilution liquid chromatography-tandem mass spectrometry (API 3200 Applied Biosystems Inc.) according to the method of Maunsell et al [26]. This method quantifies 25(OH)D_2_ and 25(OH)D_3_ including the 3-epimer form, which is not separated from 25(OH)D_3_. The limit of quantification for the assay was <5 nmol/L for both metabolites. The assay’s within-run (5% or less) and between-day imprecision (10% or less) was determined using the UTAK Tri-Level control material (UTAK Laboratories, Inc. CA, USA). Traceability was confirmed directly to National Institute of Standards & Technology (NIST) Standard Reference Material (SRM) 972 level 1–4. Levels of serum 1,25-dihydroxyvitamin D were measured by a ^125^I-based radioimmunoassay (IDS Immuno-Diagnostic Systems, Boldon, UK) with a normal reference range of 48–150 pmol/L as stated by the manufacturer. Lower limit of detection was 8 pmol/L with an assay range of approximately 7–500 pmol/L. The within-run CVs ranged from 6% to 25% and between-day CV (n = 9) was 16%. Intact PTH was determined using an automated electrochemiluminescence immunoassay (Elecsys 1010, Roche Diagnostics). Manufacturer controls (Elecsys Precision Bone 1, 2 and 3) were analysed with each reagent kit. The mean (inter-assay CV, %, n = 7) for the three controls were 55 (8.1%), 197 (1.2%) and 829.1 (1.6%) ng/L, respectively, and were within the range of results provided by the manufacturer.

### Statistical methods

Results for serum 25(OH)D, 1,25(OH)_2_D and PTH were available for 529, 523 and 518 children, respectively. All available data were used for each analysis (in some cases serum samples were insufficient for assays and missing values were considered missing completely at random). Descriptive statistics were used to summarize the data. Initially, unadjusted linear regressions were performed to assess associations between age, sex, and anthropometric factors and circulating 25(OH)D, 1,25(OH)_2_D and PTH concentrations. Adjusted linear regression analysis was subsequently performed to determine the independent contributions of demographic, biochemical and anthropometric determinants of circulating 25(OH)D and1,25(OH)_2_D concentrations. Interactions between age and sex and between BMI and sex were investigated and where 25(OH)D or 1,25(OH)_2_D were included in the model, interactions were also tested between these and sex. Log-transformations were investigated and used when the properties of the model residuals were improved in terms of normality, homoscedasticity, and linearity. Non-linearities were examined including a quadratic term, which was retained when statistically significant. Robust standard errors were used to account for clustering within each of the ten schools. All analyses were conducted using Stata 11.2 (StataCorp, College Station, TX, USA) with a 2-sided 0.05 level of significance used in all cases.

## Results

Demographic characteristics and selected anthropometric parameters of the participants are shown in **Table 1**. Of the children with 25(OH)D available, the prevalence of stunting, underweight and thinness were 10.2% (54 of 529), 17.4% (48 of 276) and 11.6% (61 of 528), respectively. No child was taking a vitamin and mineral supplement.

**Table 1:** Selected characteristics of the study sample^1^.

Variable	n	
Age, *y*	529	9.9±1.7
5 to 8 y, %	91	17.5
8 to 10 y, %	181	33.5
10+ y, %	257	49.0
Girls, %	268	50.5
Menarche, %	5	2.0
Weight, *kg*	536	26.3±1.3
Height, *cm*	537	131.5±10.4
BMI, kg/m	536	14.8 (2.2)
Weight-for-age *z* score^2^	279	−1.3 (1.1)
Height-for-age *z* score	537	−1.0 (1.2)
BMI-for-age *z* score	536	1.0 (1.2)
Serum 25-hydroxyvitamin D, *nmol/L*	529	72.7±1.2
Serum 1,25-dihydroxvitamin D, *pmol/L*	523	199.1±1.3
Parathyroid hormone, *ng/L*	518	35.0±1.5

1 Values are geometric mean ± geometric SD or median (IQR) unless specified. *Note*: Geometric SD is a factor, not a quantity. *Powers* of the geometric SD can be either multiplied by (or divided into) the geometric mean to determine the set of values that lie *n* geometric SDs from the mean.

2 WHO Reference 2007 does not provide weight-for-age charts beyond 10 years of age.

The distributions of serum 25(OH)D, 1,25(OH)_2_D, PTH values were all positively skewed and so geometric means and standard deviations are used to describe their distributions. One implausibly low value for 1,25(OH)_2_D was excluded from analysis (13 pmol/L). In the study sample as a whole, geometric mean (SD) serum 25(OH)D concentration was 72.7 (1.2) nmol/L, ranging from 36.1 to 127.0 nmol/L (**Table 1**). Only 21 (4%) participants had a serum 25(OH)D level below 50 nmol/L (20 ng/mL), of whom 4 (1% of the total sample) participants had circulating 25(OH)D concentrations below 40 nmol/L (16 ng/mL). The overall geometric means (SD) for serum 1,25(OH)_2_D and PTH concentration was 199.1 (1.3) pmol/L (range 66 to 481 pmol/L) and 35.0 (1.5) ng/L (range 7.9 to 241 ng/L), respectively.

**Figure 1:**
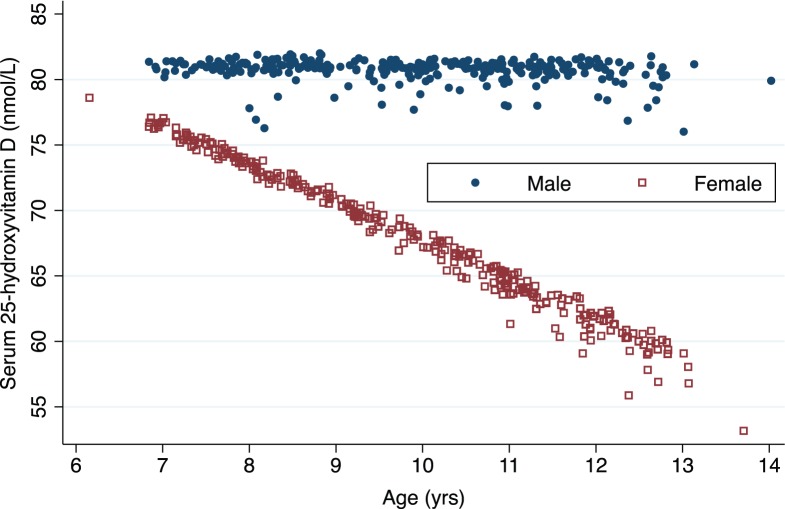
Relation between age in years and serum 25-hydroxyvitamin D (nmol/L) concentrations stratified by gender.

**Table 2** presents the geometric mean (95% CI) serum 25(OH)D, 1,25(OH)_2_D, and PTH for the children by sex and age groups. Regression analyses of the association between potential determinants (sex, age height and BMI) and serum 25(OH)D and 1,25(OH)_2_D are presented in **Table 3**. Unadjusted linear regression indicated that serum 25(OH)D was statistically significantly positively associated with height and negatively associated with age, with boys having a statistically significantly higher serum 25(OH)D than females. In adjusted analyses, there was statistically significant evidence for an interaction between sex and age with regards to 25(OH)D concentrations. Specifically, serum 25(OH)D concentrations were 19% higher in males at the mean age (9.9 years). Furthermore, there was evidence of sex differences in the association with age (P<0.001). We did not observe a decline in 25(OH)D concentrations in male participants with increasing age (P = 0.90), however, females experienced a statistically significant 4% decline in serum 25(OH)D levels for each increasing year of age (**Figure 1** **)**.

**Table 2:** Serum 25-hydroxyvitamin D, 1,25-dihyroxyvitamin D and parathyroid hormone concentrations in school-aged children by sex and age group^1^.

		Females						Males					
Variable	n	5–8 y	n	8–10 y	n	10+ y	n	5–8 y	n	8–10 y	n	10+ y	n
Serum 25(OH)D, *nmol/L*	529	74.7±1.2	52	70.1±1.2	86	61.9±1.2	130	80.4±1.2	39	78.8±1.2	94	79.4±1.2	128
Serum 1,25(OH)_2_D, *pmol/L*	523	213.0±1.3	52	203.1±1.3	81	221.7±1.3	129	184.1±1.3	39	182.7±1.3	93	183.4±1.5	129
Serum PTH, *ng/L*	518	36.6±1.4	51	35.8±1.5	81	42.3±1.5	127	31.2±1.5	39	29.4±1.5	93	33.0±1.5	127

1 Values are geometric mean ± geometric SD. *Note:* Geometric SD is a factor, not a quantity. Powers of the geometric SD can be either multiplied by (or divided into) the geometric mean to determine the set of values that lie *n* geometric SDs from the mean.

**Table 3:** Linear regression model of factors associated with serum 25-hydroxyvitamin D (nmol/L), 1,25-dihydroxyvitamin D (pmol/L) and parathyroid hormone concentrations (ng/L) among Thai schoolchildren aged 6–13 y^1^.

	Unadjusted				Adjusted/final^2^^,^ 3			
Variable	n	Estimates	95% CI	*P*	n	Estimates	95% CI	*P*
For 25(OH)D					525			
BMI	525	0.99	0.98, 1.00	0.021		0.997	0.987, 1.01	0.515
Sex (male)	526	1.19	1.14, 1.23	<0.001		1.19 4	1.14, 1.23	<0.001
Age, y	526	0.98	0.96, 0.99	0.010				
Age, y for boys						1.00	0.98, 1.02	0.895
Age, y for girls						0.96	0.94, 0.97	0.001
Sex × Age interaction								<0.001
Height-for-age Z score	526	0.97	0.94, 0.99	0.017		0.98	0.96, 0.99	0.006
For 1,25(OH)_2_D					510			
BMI	518	1.03	1.00, 1.06	0.028		1.11	1.04, 1.18	0.004
BMI-squared		0.996	0.994, 0.999	0.009		0.997	0.996, 0.999	0.004
Sex (male)	519	0.86	0.83, 0.89	<0.001		0.86	0.82, 0.90	<0.001
Age, y	519	1.02	0.99, 1.05	0.232		1.02	0.99, 1.04	0.17
Height-for-age Z score	519	1.07	1.05, 1.110	<0.001		1.07	1.04, 1.11	0.001
log 25OHD	512	0.91	0.76, 1.08	0.235		1.12	0.95, 1.33	0.153
For PTH					502			
BMI	514	1.02	0.99, 1.04	0.196		0.98	0.96, 1.02	0.365
Sex (male)	515	0.80	0.74, 0.87	<0.001		0.91	0.82, 1.02	0.097
Age, y	515	1.04	1.01, 1.07	0.008		1.03	0.999, 1.07	0.055
Age-squared		1.02	1.00, 1.04	0.037		1.02	1.00, 1.03	0.049
Height-for-age Z score	515	1.10	1.03, 1.18	0.012		1.08	1.02, 1.14	0.016
log 25OHD	509	0.50	0.43, 0.57	<0.001		0.61	0.49, 0.74	<0.001
log 1,25(OH)_2_D	509	1.34	1.09, 1.64	0.012		1.18	0.99, 1.40	0.06

1 Effect sizes are ratios of geometric means, accompanied by 95% CIs and *P*-values.

2 Adjusted for variables with *P*≤0.25 in univariate analysis.

3 R^2^ of the whole model for 25OHD: 0.24; for 1,25(OH)_2_D: 0.13; for PTH: 0.20.

4 At the mean age (9.9 y).

Unadjusted linear regression indicated that serum 1,25(OH)_2_D was statistically significantly positively associated with height-for-age Z-score (**Table 3**). In contrast to 25(OH)D, boys had a statistically lower serum 1,25(OH)_2_D than females. There was also evidence of an n-shaped association with BMI, with 1,25(OH)_2_D levels peaking around 19.9 pmol/L. All of the above relations remained statistically significant in the adjusted model. For PTH, females also appeared to have a statistically significantly higher serum PTH in the unadjusted model, although this effect was attenuated (approximately halved) in the adjusted model and was no longer statistically significant (P = 0.10). As expected, serum 25(OH)D remained statistically significantly negatively associated with serum PTH concentrations in the adjusted model and height remained significantly positively associated with PTH. For every one unit increase in height-for-age Z score, 1,25(OH)_2_D and PTH concentrations increased by 7% and 8%, respectively. Stunting (height-for-age Z-score<−2 SD, *n* 54 children) was not associated with either 1,25(OH)_2_D or PTH concentrations (data not shown).

## Discussion

To our knowledge, this is the first study in Thailand to evaluate differences in 25(OH)D status with age and to directly compare boys with girls in a large sample of healthy school aged children. Using cut-offs recommended by the U.S. Institute of Medicine (IOM) [serum 25(OH)D <50 nmol/L] [27], the present study results indicate that only 4% of the children were vitamin D deficient and PTH concentrations were within normal range. The low prevalence of vitamin D deficiency in our data contradicts recent findings demonstrating 30–52% prevalence of deficiency among healthy school-aged children from different regions of the country [28]. Several other studies in various clinical pediatric groups including asthmatic and thalassemia Thai patients [29], [30] have also reported a high prevalence of vitamin D deficiency, although these selected patient groups are known to be predisposed to the development of vitamin D deficiency. Potential reasons for the discrepancy between our finding and the substantially higher prevalence recently reported include differences in the analytical measurement employed. In our study, serum 25(OH)D concentrations were measured using isotope dilution LC-MS/MS, which tends to have higher sensitivity and selectivity, and consequently often reports higher values over various automated assays [31], [32]. However, it is unlikely to be the only reason as geographical location could also contribute to differences in vitamin D status. Data from the Thai National Health Examination Survey demonstrated higher 25(OH)D levels in adults in the northern than in southern regions of the country in addition to lower vitamin D status among those residing in Bangkok, where pollution was higher [22]. Children in the present study were sampled from a single geographical region – the northeast. Consistent with our findings, the prevalence of vitamin D deficiency among Thai adults residing in the northeast region was less than 3% and low serum 25(OH)D levels were associated with being female with mean 25(OH)D levels being approximately 10 nmol/L higher in males [22].

The inverse association of serum 25(OH)D levels with age found only among female participants may reflect reduced sun exposure, however, no measurement of UVB exposure or time spent outdoors was collected in our study. A 2010 survey of sun protection behavior among adolescents in Bangkok demonstrated that females had significantly higher adoption of sun protection behavior compared to males including increase use of sunscreen, staying under the shade and use of umbrellas [33].

Serum concentrations of 1,25(OH)_2_D in healthy populations have not been well described. In the current study, 1,25(OH)_2_D concentrations in our sample of Thai children (ranging from an average of 190 and 210 pmol/L among males and females, respectively) were approximately 50 pmol/L higher than those reported in U.S. children [6], [34], [35]. Serum 1,25(OH)_2_D concentration is regulated based on its need in calcium homeostasis [36]. A low dietary calcium intake markedly increases serum 1,25(OH)_2_D [37] and therefore, the differences in 1,25(OH)_2_D concentrations may be due to the relatively low calcium intakes of our children compared with those of Western counterparts. While dietary intake data were not collected at study endline, baseline median calcium intakes of a randomly selected subsample of our study children (n = 230) were 222 mg/d, as reported previously [20]. Moreover, we found no statistically significant differences in reported calcium intakes between males and females at baseline (regression adjusting for clustering and following log transformation, *P* = 0.85; data not shown).

While there are no data evaluating serum 1,25(OH)_2_D levels in younger population groups with characteristically low calcium intakes, the higher concentration of 1,25(OH)_2_D observed in our study was similar to those reported in rural Gambian women with habitually low calcium intakes and sufficient vitamin D status [38]. It has been postulated that the compensatory increased production of 1,25(OH)_2_D due to low calcium intakes increases the catabolism of 25(OH)D [14]–[18]. We, however, found no overall relationship between 25(OH)D and 1,25(OH)_2_D concentrations. This is not unexpected as the circulating levels of 1,25(OH)_2_D are tightly regulated by gene expression of 1-α-hydroxylase (CYP27B1) and not by the concentration of 25(OH)D. Instead, concentrations of 1,25(OH)_2_D showed a positive association with serum PTH, which is consistent with the well-known effect of PTH to upregulate CYP27B1 and increase the conversion of 25(OH)D to 1,25(OH)_2_D in the kidneys [39]. Nonetheless, the existing high serum 25(OH)D status of the study children limits the possibility of indirectly detecting enhanced catabolism of serum 25(OH)D. Additional studies in population groups with lower concentrations of serum 25(OH)D and a direct measurement of 24,25-dihdroxyvitamin D [first metabolic product of 25(OH)D] are needed.

Female children in the current study had significantly higher concentrations of 1,25(OH)_2_D than male children. Similar findings of a sex difference in 1,25(OH)_2_D levels are supported by two previous studies [6], [34]; however, there is little information available on the influence of gender on vitamin D and calcium homeostasis in this life-cycle group. It has been reported that estrogen replacement therapy increases plasma concentration of 1,25(OH)_2_D by stimulating renal 1,25(OH)_2_D production [40]. Although sexual maturation is associated with increasing levels of sex steroids, pubertal staging of participants was not assessed. In Thailand, the onset of puberty in girls has shifted toward a younger age over the last 20 years, with the mean age at menarche of 12.1 years [41]. In our study, puberty was likely not a significant confounder as menarche had commenced in only 2% (5 of 262) of the female participants studied with 1,25(OH)_2_D values available.

The significance of the n-shaped association between 1,25(OH)_2_D and BMI, where participants in the lowest and highest BMI groups displayed lower 1,25(OH)_2_D concentrations, is unclear and warrants further investigation over a wider range of body mass indices. Very few children in our sample were overweight (n = 17) or obese (n = 13). Several studies have reported an inverse relationship between vitamin D status and childhood body weight [42]–[45]; however we found no association between 25(OH)D and BMI.

The present data also suggest a positive association between vitamin D metabolites and child height. Few studies have investigated the relationship between vitamin D and linear bone growth in healthy children. An observational study by Lund et al reported a positive association between 1,25(OH)_2_D and growth velocity in a group of children and adolescents ranging in age from 3 months to 19 years [46]. Currently, there is no simple explanation for this finding; however, there is evidence that suggests a role of the vitamin D endocrine system in regulating growth of the skeleton [47]–[50]. There is also an interrelation between vitamin D and insulin-like growth factor-1 (IGF-1), an important regulator of skeletal growth and development, albeit the relationship is complex [50], [51]. Further studies are required to confirm these findings and establish the mechanisms by which these effects are induced.

Interestingly, while a strong negative association between serum levels of 25(OH)D and PTH was demonstrated, PTH concentrations were within a normal range despite possible low calcium intakes. These results support a similar study in adults where adequate vitamin D status ensured ideal PTH values even when calcium intakes were moderately low [52]. The authors concluded that vitamin D may have a calcium-sparing effect [52]. Although the WHO recommends calcium intakes of 700 mg/d for children 7–9 years of age and 1300 mg/d for children 10–18 years [53], it is increasingly recognized that calcium requirements may be lower for Southeast Asian and East Asian populations [54], [55]. A number of dietary factors including phytate, sodium and animal protein, are known to influence the bioavailability of calcium for absorption and/or urinary calcium excretion. In the case of vitamin D, it has been long recognized that vitamin D promotes calcium absorption [56], however, the relation between vitamin D status and intestinal calcium absorption in settings of habitual low calcium intake has not been extensively studied.

This study has several notable strengths including the fairly large sample size of healthy female and male children across a wide age range. Nonetheless, our results are not necessarily generalizable to other geographical regions or population groups. These findings, however, emphasize the need for nationally representative data to appropriately assess the magnitude of vitamin D deficiency, particularly given that our study data are over 10 years old and lifestyle characteristics may have changed including sun exposure protection practices or levels of outdoor activity. Moreover, dietary calcium intakes of participants were not assessed at the time of blood sampling (i.e., study endline; only determined at study baseline). As mentioned above, in order to address the speculation that 25(OH)D concentrations are mediated by the effects of 1,25(OH)_2_D, additional studies with careful dietary assessment over a wider range of both calcium intakes and vitamin D status are needed. In doing so, careful consideration should also be given to the type of assay employed to measure 1,25(OH)_2_D as accurate measurement of this vitamin D metabolite is particularly difficult [57].

In summary, our results suggest that vitamin D status was sufficient in this group of Thai school children, although girls experienced a decline in 25(OH)D levels with increasing age. This association is possibly mediated by reduced sun exposure. Future studies should employ measures to appropriately quantify sun exposure and outdoor activity. In addition, this study is the first to investigate the interrelationship between vitamin D metabolites in a group of healthy children who typically have low calcium intakes. While the increased concentration of 1,25(OH)_2_D may reflect an adaptive mechanism to maximize calcium absorption, more extensive studies are needed to define the ideal intake of calcium in the presence of an ample supply of vitamin D.
